# Alp7/TACC-Alp14/TOG generates long-lived, fast-growing MTs by an unconventional mechanism

**DOI:** 10.1038/srep20653

**Published:** 2016-02-11

**Authors:** Frauke Hussmann, Douglas R. Drummond, Daniel R. Peet, Douglas S. Martin, Robert A. Cross

**Affiliations:** 1 Warwick Medical School, Coventry CV4 7AL UK

## Abstract

Alp14 is a TOG-family microtubule polymerase from *S. pombe* that tracks plus ends and accelerates their growth. To interrogate its mechanism, we reconstituted dynamically unstable single isoform *S. pombe* microtubules with full length Alp14/TOG and Alp7, the TACC-family binding partner of Alp14. We find that Alp14 can drive microtubule plus end growth at GTP-tubulin concentrations at least 10-fold below the usual critical concentration, at the expense of increased catastrophe. This reveals Alp14 to be a highly unusual enzyme that biases the equilibrium for the reaction that it catalyses. Alp7/TACC enhances the effectiveness of Alp14, by increasing its occupancy. Consistent with this, we show in live cells that Alp7 deletion produces very similar MT dynamics defects to Alp14 deletion. The ability of Alp7/14 to accelerate and bias GTP-tubulin exchange at microtubule plus ends allows it to generate long-lived, fast-growing microtubules at very low cellular free tubulin concentrations.

In eukaryotic cells, networks of microtubules (MTs) serve as dynamic scaffolds and railways for the organisation of the cell contents. Within MT networks, individual MTs typically show dynamic instability, an energy-dissipating cycle of growth, catastrophe, shrinkage, rescue and regrowth that is driven by GTP turnover. Dynamic instability is an intrinsic property of MTs built only from pure tubulin, which in cells is typically controlled and harnessed by microtubule associated proteins (MAPs). MAPs interact either with the newly-polymerised GTP-caps of microtubules^1^, or with their GDP-lattice, or both. Amongst proteins that modulate dynamic instability, TOG-family MT polymerases are of central importance^2^. Alp14, a TOG family polymerase from *S. pombe,* has a plus-end specific^3^ action that is essential for the normal dynamics of mitotic and meiotic spindles^4^ and of interphase MTs^5^.

Alp7 (Mia1p), a TACC (transforming acidic coiled coil) protein, is a binding partner for Alp14^6^ that plays multiple, critical roles in cell division. Alp7 is required for nuclear retention of Alp14 and for recruitment of Alp14 to *S. pombe* kinetochores^7^. Alp7 recruits Alp14 early in mitosis to spindle poles bodies^8^ and also targets Alp14 to kinetochores via its binding to NDC80^9^. Alp7 is imported into the nucleus under the control of the Ran-GTPase^10^, recruits a Klp5/6-PP1 complex to kinetochores^11^, is required for the maintenance of microtubules at spindle pole bodies^12^, is involved in MT crosslinking in mitosis^13^, and is a key target for mitotic kinases^14^. These observations are all consistent with a role for Alp7 as a targeting subunit for Alp14. Whether Alp7 binding influences the polymerase mechanism of Alp14 is not clear.

To date, most work on the molecular mechanisms of TOG-family MT polymerases has focused on XMAP215^2^,^15^, a monomer with five TOG domains that controls the total MT mass in *Xenopus* spindles^16^. Alp14 is composed of two identical chains, each with two TOG domains connected to a C-terminal tail, like its *S. cerevisae* homolog, Stu2^17^. It was initially shown that XMAP215 binds and stabilises short MT protofilaments (PFs)^18^,^19^ and XMAP215 was proposed to shuttle these into the growing tip^20^. Subsequently, single molecules of GFP-XMAP215 were found to be processive at MT tips, ruling out PF shuttling as the main pathway of growth, and indicating instead a catalytic model in which the polymerase accelerates the exchange of individual GTP-tubulin subunits at plus ends^21^.

Recent work on Alp14^3^ acting on brain tubulin *in vitro* found complex behavior, with accelerated plus end growth at lower concentrations of Alp14 and inhibited plus end growth at high concentrations of Alp14. To add to the potential complexity, work on Stu2 has shown it to be a weak polymerase of porcine brain tubulin but a strong polymerase of *S. cerevisae* tubulin^22^. TOG-family polymerases are reported to alter the dynamics of MT catastrophe: Stu2 inhibits catastrophe^22^; XMAP215 has mixed effects^22^; whilst Alp14 is reported to increase catastrophes on brain tubulin^3^. Understanding the mechanisms of these various effects is an important challenge.

Dissecting the mechanism by which TOG-family polymerases influence MT dynamics requires that we understand the underlying dynamic instability mechanism of pure tubulin. Some aspects of this are now clear. The fundamental building blocks for MTs are GTP-tubulin heterodimers. Above a critical concentration, GTP-tubulin heterodimers add steadily to the growing tips of MTs. GTP hydrolysis follows, and the GDP-tubulin subunits produced try to adopt a bent conformation that is incommensurate with the straight MT lattice^23^. The resulting mechanical strain in the GDP-tubulin core of the MT is the driver for dynamic instability, whereby steady MT growth only continues for as long as the stabilising cap of recently-incorporated GTP-tubulins remains intact. The terminal layer of the GTP cap is robust against hydrolysis, because the GTPase active site at the plus end of each PF is not hydrolysis-competent until a further GTP-tubulin subunit binds^24^. Depletion of the GTP-tubulin cap by hydrolysis or GTP-tubulin dissociation can breach the stabilizing GTP cap^25^, causing catastrophe and depolymerisation.

The detailed mechanism of MT growth is important for models of TOG family polymerase mechanism. In one current model, growing PFs incorporate GTP-tubulin very rapidly, almost at a diffusion-limited rate, but then usually undergo a mini-catastrophe^26^. In a more classical view, GTP-tubulin addition and loss are both relatively slow^27^. In the first case, for TOGs to boost net MT growth they would only need to stabilise the nascent PFs. In the second case, TOGs would need to catalyze the addition of GTP-tubulin, for example by enriching an exchange intermediate of GTP-tubulin^21^. Recent X-ray structures of *S. cerevisae* Stu2 TOG1 and 2 domains complexed to *S. cerevisae* tubulin reveal possible structures for a TOG-stabilised GTP-tubulin reaction intermediate^28^,^29^. The structures are very similar, with the tubulin in both complexes bent to an extent that would be incompatible with full incorporation into the MT lattice. Potentially, stabilising a partially-bent reaction intermediate could allow TOG-family polymerases to catalyse both the landing and leaving reactions of GTP-tubulin.

In this work we have studied the polymerase mechanism of Alp14 on *S. pombe* microtubules, and the influence of Alp7 on this mechanism. We show that Alp14 is a highly unusual enzyme that increases the on- and off-rate constants for GTP-tubulin to different extents, thereby shifting the overall equilibrium constant for GTP-tubulin exchange at plus ends and lowering the effective critical GTP-tubulin concentration for plus end growth. Further, we show that Alp7 binding potentiates the polymerase activity of Alp14, both *in vitro* and *in vivo*.

## Results

### Alp14 and Alp7 are both required for accelerated microtubule assembly *in vivo*

**Figure 1:**
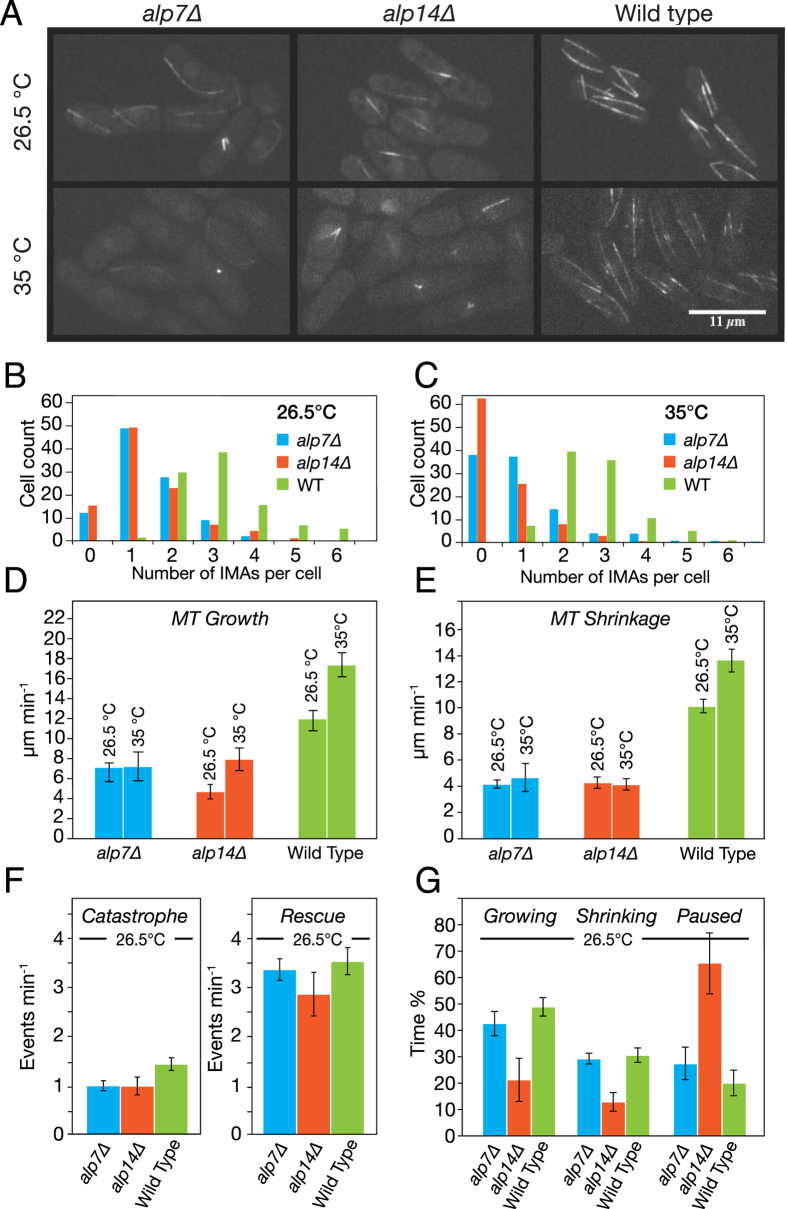
*In vivo* effects of *alp14* deletion versus *alp7* deletion (**A**) Spinning disc microscopy of GFP-labelled MTs in control, *alp7* and *alp14* deletion strains of *S. pombe*. (**B,C**) Deletion of *alp7* or *alp14* significantly reduces the number of interphase microtubule arrays (IMAs) in each cell, at both 26.5 °C and 35 °C (Kruskal-Wallis p < 0.0001, Dunn’s post test p < 0.001)). See Movie S1. (**D**) MT growth is inhibited by *alp7* or *alp14* deletion. Mean MT growth rates are significantly different at both 26.5 °C (ANOVA, p < 0.0001) and 35 °C (p < 0.0001). Wild type is significantly faster than either *alp7* or *alp14* deletions at both 26.5 °C (p < 0.001) and 35 °C (p < 0.001). *alp7* MT growth rate in *alp7* deletions is not significantly different from that of *alp14* deletions at 26.5 °C (p < 0.001) or 35 °C (Tukey post test, p < 0.001). (**E**) MT shrinkage is inhibited by *alp7* or *alp14* deletion. Shrinkage rates are significantly different at 26.5 °C (ANOVA, p < 0.0001) and 35 °C (p < 0.0001). Wild type is significantly faster than either *alp7* or *alp14* deletions at both 26.5 °C (p < 0.001) and 35 °C (Tukey post test, p < 0.001). *alp7* deletion is not significantly different from alp14 deletion at 26.5 °C (p < 0.001) and 35 °C (p < 0.001). (**F**) Catastrophe and rescue are not significantly different in any of the genotypes, at either 26.5 °C or 35 °C (26.5 °C shown; ANOVA, respectively, p = 0.0784 and p=0.3615). (**G**) *alp14* deletion but not *alp7* deletion increases pausing. Growth times are significantly different (ANOVA, p = 0.0102). MTs in wild type cells spend significantly longer in growth than those in *alp14* deletions at p < 0.05. Differences are not significant (p < 0.05) between wild type and *alp7* deletions or between *alp14* and *alp7* deletions (Tukey post test). Times spent in shrinkage are significantly different (ANOVA, p = 0.0006). MTs in wild type and *alp7* deletions spend significantly longer in shrinkage than those in *alp14* deletions (p < 0.01). Differences between wild type and *alp7* deletions are not significant at (Tukey post test, p < 0.01). Percentage times spent paused are significantly different (ANOVA, p = 0.0022). The percentage of total time MTs spend paused is significantly longer in *alp14* deletions than in wild type or *alp7* deletions (p < 0.05). Differences between wild type and *alp7* deletions are not significant (Tukey post test, p < 0.05). Error bars are SEM.

To probe the functional relationship of Alp14 and Alp7 *in vivo*, we compared GFP-labeled interphase MT arrays (IMAs^30^) in an *alp14* deletion strain of *S. pombe* with those in an *alp7* deletion strain (Fig. 1A, Supplementary Movie 1) at both the permissive (26.5 °C) and restrictive (35 °C) temperatures for proliferation of these strains^30^. Both strains were kind gifts of Takashi Toda. In *alp14* deletes, the number of IMAs (Fig. 1B,C) per cell is reduced, in close agreement with previous work^3^. We find that *alp7* deletion produces very similar effects to *alp14* deletion at the restrictive and permissive temperatures, suggesting that the temperature sensitive process in these deletion strains occurs during mitosis. To further compare phenotypes, we tracked MT plus ends. In both deletion strains, the mean growth rate of interphase MTs is reduced by 2- to 3-fold (Fig. 1D) compared to wild type, consistent with an Alp7-14 complex being required for full polymerase activity *in vivo*. Shrinkage rates are also reduced by both deletions (Fig. 1E), raising the possibility that *in vivo* and in context, Alp7-14 complexes might accelerate shrinkage, as well as growth. Deleting *alp14* or *alp7* has little effect on catastrophe or rescue frequencies (Fig. 1F), but *alp14* deletion does clearly cause MTs to spend more time in a paused state (Fig. 1G). The requirement of Alp7 for full Alp14 activity is consistent with the view that Alp7 is a targeting factor for Alp14, but might also indicate a role for Alp7 in the polymerase mechanism of Alp14.

### Alp7/TACC binds tightly to Alp14/TOG and the complex binds GDP- and GTP-tubulin

**Figure 2:**
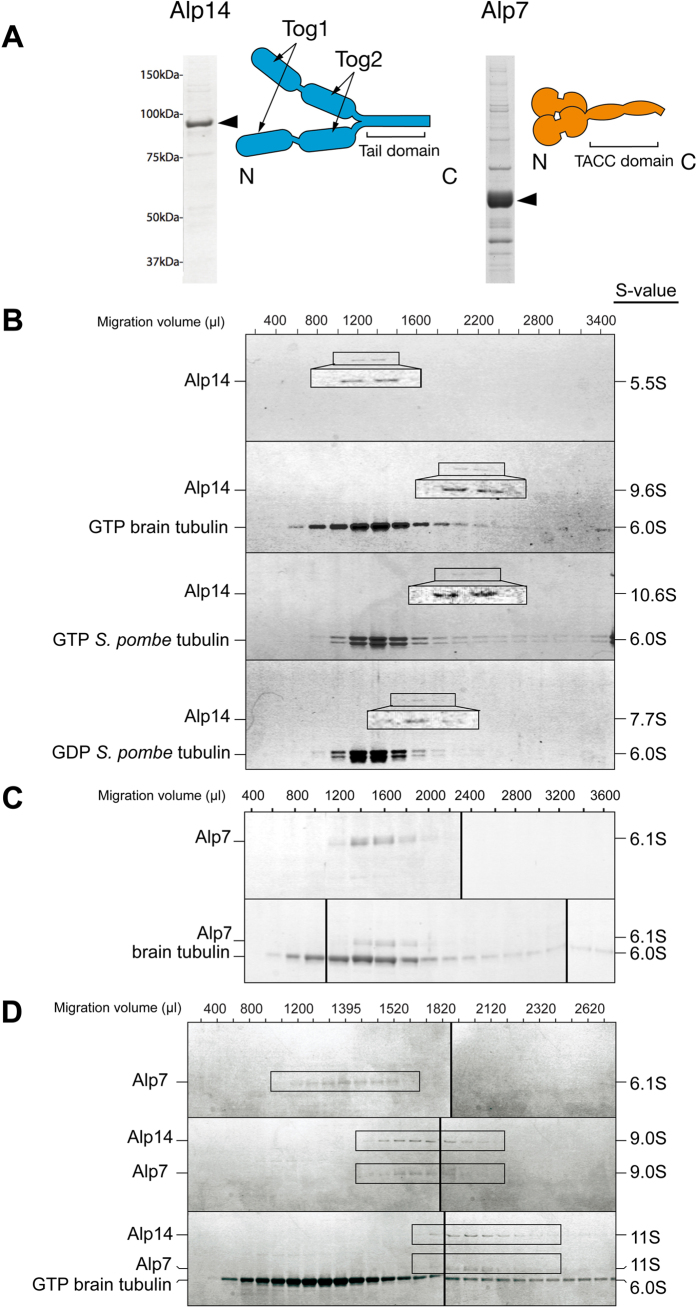
Binding interactions of *S. pombe* Alp14, Alp7 and tubulin (**A**) Full length Alp14 with a C-terminal his or Avi tag and Alp7 with an N-terminal avi-tag were expressed in a baculovirus system and purified using tag-affinity chromatography (see Methods). (**B–D)** 5–40% glycerol gradient centrifugation of purified Alp14, Alp7 and tubulin (see Methods), SDS-PAGE with Coomassie staining. (**B**) Alp14 alone and with GTP-brain tubulin and GTP and GDP *S. pombe* α1β tubulin. Peak fractions are contrast-enhanced for clarity. (**C**) Alp7 is not shifted by adding tubulin. (**D**) Alp14 is shifted further by GTP-tubulin than by GDP-tubulin. Alp7 is shifted by Alp14 and the complex is further shifted by GTP-tubulin.

To probe the potential mechanistic role of Alp7, we reconstituted Alp714-driven microtubule assembly *in vitro*. We expressed and purified full length Alp14 and Alp7 using a baculovirus system (Fig. 2A) Purified full length *S. pombe* Alp14 protein migrates at 5.5S in glycerol gradient centrifugation (Fig. 2B), in agreement with previous work and consistent with its being an extended molecular dimer. Brain tubulin binds relatively stably to Alp14 (see later) to form a 9.6S complex. Mixing *S. pombe* GTP- tubulin with full length Alp14 creates a 10.6S complex, whilst mixing Alp14 with GDP-tubulin creates a 7.7S complex (Fig. 2B), suggesting either that Alp14 binds more weakly to GDP tubulin than to GTP tubulin, or that the Alp14-GDP-tubulin complex and the Alp14-GTP-tubulin complexes have different conformations. At present we cannot distinguish these possibilities. Purified full length Alp7 migrates at 6.1S, consistent with its predicted extended dimer structure (Fig. 2C). Under our conditions, Alp7 does not bind detectably to free tubulin (Fig. 2C), which migrates at 6.0S. Mixing full length Alp14 with full length Alp7 creates a tight 9.0S complex (Fig. 2D), whilst addition of tubulin creates an 11.0S Alp7/14-tubulin complex (Fig. 2D). We conclude that Alp14 and Alp7-14 bind free tubulin, but that Alp7 alone does not.

### Alp14 specifically tracks the plus ends of *S. pombe* MTs and accelerates their growth

**Figure 3:**
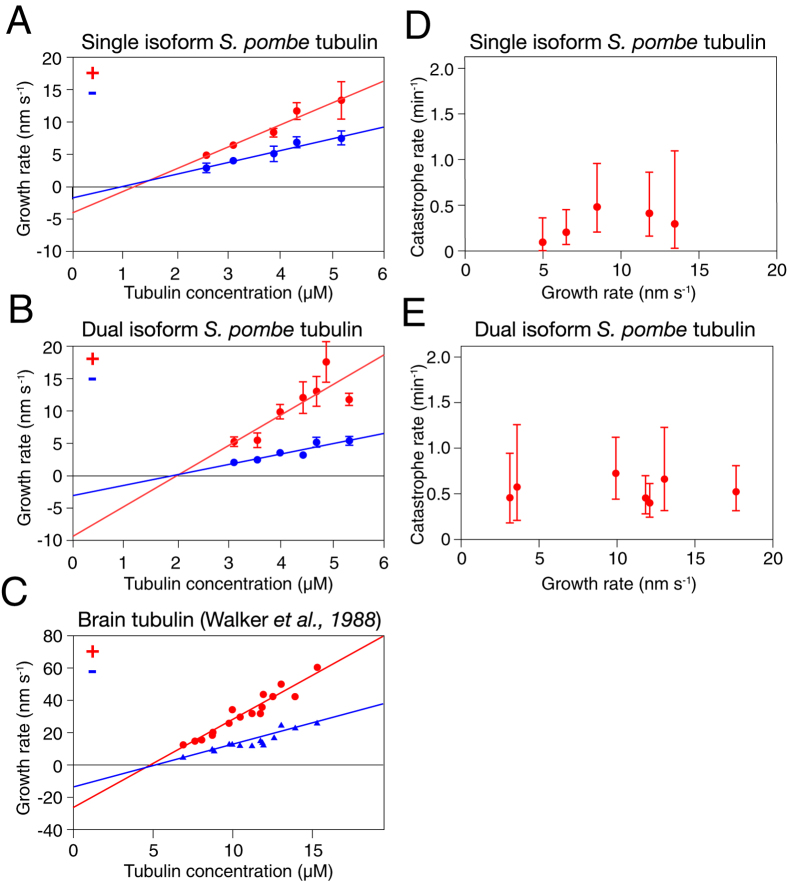
*In vitro* growth and catastrophe of *S. pombe* MTs Plus end (red) and minus end (blue) growth rates versus tubulin concentration for purified *S. pombe* single (α1β) (**A**) and dual-isoform (α1β + α2β) tubulin (**B**), measured by DIC video microscopy at 25 °C. Tubulin concentrations were corrected for variable (minor) amounts of assembly-incompetent tubulin, quantitated using a MT pelleting assay. Dual isoform tubulin contains approx. 50:50 α1β and α2β tubulin heterodimers. Conditions: 100 mM K-PIPES, 1 mM MgSO_4_, 2 mM EGTA, 1 mM DTT pH 6.9, no glycerol, 25 °C. Measured rate constants are summarized in Table 1. Similar data replotted from Walker *et al.*^27^ for mammalian brain tubulin at 37 °C (**C**) Catastrophe rates versus MT growth rate for (**D**) α1β and (**E**) α1β + α2β tubulin. Error bars are SEM (**A–C**) and 95% CI (**D,E**).

Previous work on Alp14 has used brain tubulin. To examine the effects of Alp14 and Alp7 on *S. pombe* MTs, we began by measuring rate constants for the basal dynamic instability of MTs assembled from purified *S. pombe* tubulin. We purified untagged single and dual isoform tubulins from S. pombe by ion exchange chromatography, assembly-disassembly and a final gel filtration step (see Methods), assembly-disassembly and a final gel filtration step (see *Methods*). We followed dynamic instability using video-enhanced DIC microscopy^27^. As shown in Fig. 3, the plus and minus end growth rates of *S. pombe* single and dual-isoform microtubules vary linearly with tubulin concentration (see Table 1 for rate constants). For a simple bimolecular assembly mechanism where growth rate = k_on_ [tubulin] − k_off_^27^ the slopes of these plots (Fig. 3A,B) give the apparent second order rate constants for GTP-tubulin binding at the plus and minus ends of the MT and the Y-axis intercepts define the apparent first order rate constants for GTP-tubulin dissociation at each end. Data from Walker *et al.*^27^ for brain tubulin are shown for comparison (Fig. 3C). We also measured the tubulin concentration dependence of MT catastrophe. Within the accessible tubulin concentration range, the catastrophe rate appeared independent of tubulin concentration and MT growth rate, for both single isoform and dual isoform *S. pombe* MTs (Fig. 3D,E; Table 1).

**Figure 4:**
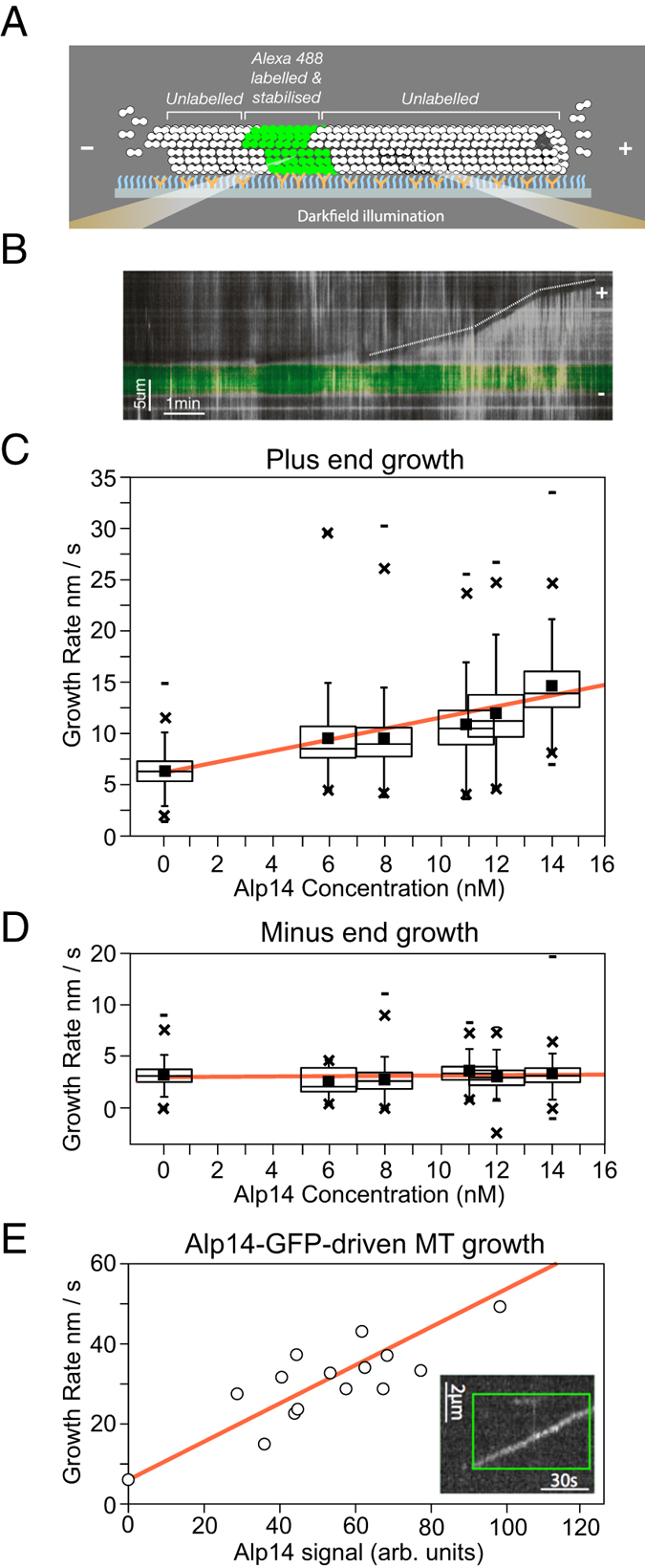
Alp14 tip tracks at plus ends of *S. pombe* MTs and accelerates their growth (**A**) Schematic of experiment. MTs were grown by polymerising unlabeled single isoform *S. pombe* tubulin on to stabilised fluorescently labelled seeds that were tethered to the coverslip using anti-fluorophore antibodies. Dynamics were visualized using darkfield video microscopy. Darkfield illumination was done at a wavelength that did not excite the fluorophore (see Methods). (**B**) Example darkfield data. The dotted overlay highlights infrequent transitions that occur between phases of linear growth. The green band in the kymographs is the fluorescent MT seed. (**C**) Mean plus end growth rate of *S. pombe* MTs increases linearly with increasing Alp14 concentration. (**D**) Minus end growth rate is unaffected. (**E**) Alp14-GFP tip tracks on unlabeled *S. pombe* MTs (*Inset)* MT growth rate increases with increasing Alp14-GFP occupancy of MT tips.

To examine the effects of Alp14 on dynamic instability, we used darkfield microscopy^31^ to image the assembly of unlabeled *S. pombe* tubulin (Fig. 4A) on to fluorescently labeled, stabilised MT seeds. Adding full length Alp14 to dynamic single isoform *S. pombe* MTs increases their plus end growth rate (Supplementary Movie 2). Kymographs reveal that Alp14-driven growth is episodic, with MTs growing at a constant rate for several tens of seconds and then switching to a higher or lower growth rate, which is then again typically maintained for several tens of seconds (Fig. 4B). At the low-nanomolar concentrations of Alp14 that we assayed, mean MT growth rate increases roughly linearly with Alp14 concentration (Fig. 4C), with no effect on minus end growth (Fig. 4D). Assays at higher Alp14 concentrations were not feasible, because Alp14 enhances the nucleation of *S. pombe* MTs in solution so much that the field becomes intractably crowded. Following a shrinkage event, the rate of MT regrowth from seeds was independent of the previous growth rate (not shown), suggesting Alp14 dissociates before shrinkage. We suspect the episodic growth we observe reports the loading and unloading of Alp14 molecules, with both loading and unloading occurring at ~1 min^−1^ under our experimental conditions. Using Alp14-GFP allowed us directly to confirm Alp14 tip tracking. Alp14-GFP increases the plus end growth rate of *S. pombe* MTs, with, under our conditions, a linear dependence of growth rate on the intensity of the Alp14-GFP signal at the growing tip (Fig. 4E).

### Alp14 drives plus end growth at ordinarily-subcritical tubulin concentrations, at the expense of increased catastrophe

**Figure 5:**
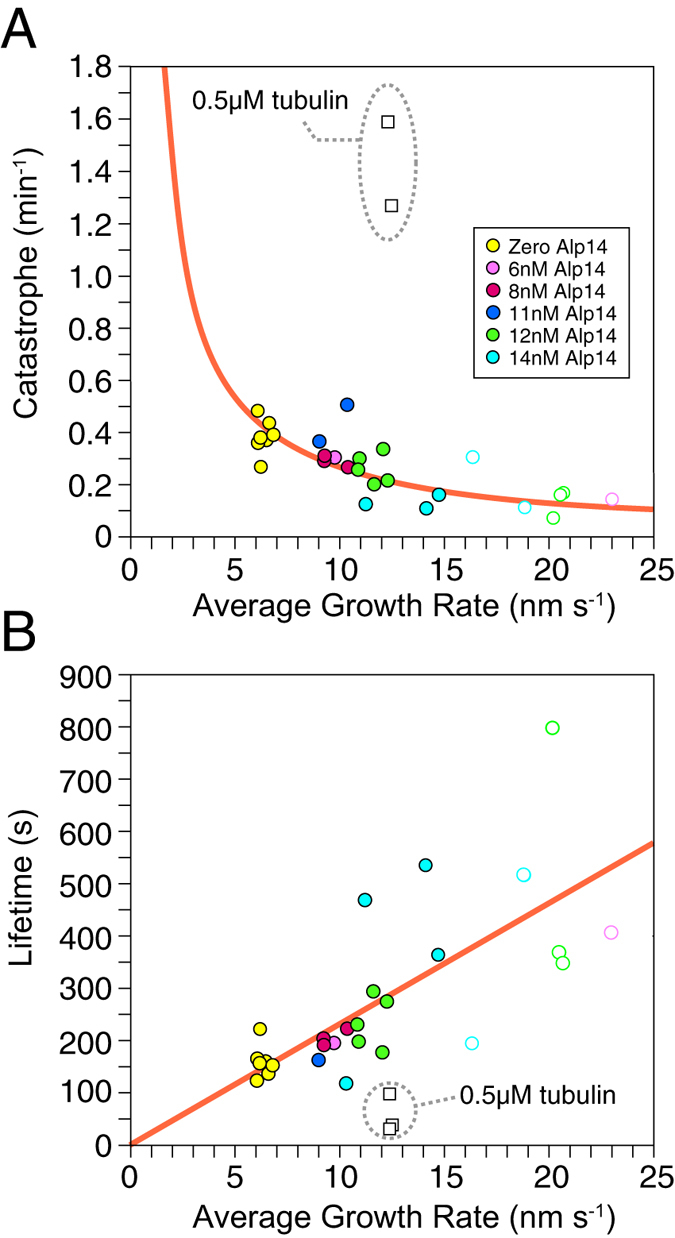
Alp14-driven growth rates of *S. pombe* MTs versus catastrophe rate and lifetime Two plots of the same data to show (**A**) Catastrophe rate and (**B**) Lifetime versus Alp14-driven MT growth rate. Filled data points show the mean plus end growth rate of individual MTs growing from surface-immobilized GMPCPP-stabilised MT seeds, with increasing growth rates driven by increasing concentrations of tip-tracking Alp14 as in Fig. 4C. The ambient concentrations of Alp14 used in the assays are colour coded. There is only a loose correlation of growth rate to ambient Alp14 concentration, because there is a large variation in the amount of Alp14 that loads to individual MTs. Open data points at high growth rates are from MTs that nucleated *de novo* during the assays. All data obtained at 6 μM tubulin, except for the open squares data points (encircled) which were obtained by reducing the tubulin concentration to 0.5 μM and increasing Alp14 concentration sufficiently to maintain growth at ~12 nm s^−1^. Under these conditions, the growth rate is maintained at the expense of excess catastrophes.

At 6 μM GTP-tubulin, MTs assembled from *S. pombe* tubulin exhibit full dynamic instability, growing steadily, then catastrophising, shrinking back to their seed and regrowing. Increasing the MT plus end growth rate by adding Alp14 decreased the plus end catastrophe rate (Fig. 5A), with no effect on minus ends (not shown). MT lifetime (the inverse of the catastrophe rate) increased linearly (orange fit) with the rate of Alp14-catalysed MT plus end growth (Fig. 5B). To probe the assembler mechanism of Alp14, we lowered the tubulin concentration whilst increasing the Alp14 concentration sufficiently to maintain the plus end growth rate. Ultimately, we were able to reduce the GTP-tubulin concentration to 0.5 μM, whilst maintaining steady growth at 12 nm s^−1^, equal to the growth rate measured at 6 μM GTP-tubulin in the absence of Alp14. Importantly, this increased the catastrophe rate. With 0.5 μM GTP-tubulin and sufficient Alp14 added to maintain growth at 12 nm s^−1^, the catastrophe rate was 5-fold higher than at 6 μM GTP-tubulin in the absence of Alp14 (Fig. 5A). We conclude that Alp14 lowers the effective critical GTP-tubulin concentration for plus end growth, but at the expense of increased catastrophe.

### Alp14 promotes the nucleation of new MTs

Besides accelerating the plus end growth rates of *S. pombe* MTs, Alp14 also promotes the nucleation of fresh MTs. In our reconstitution assays, stabilised MT seeds are bound to the coverslip surface using anti-fluorophore antibodies and the coverslip is then passivated with Tween 20 (see Methods). In these assays in the presence of Alp14, new MTs form in solution, land on the Tween-20 passivated surface and continue to grow. These MTs grow faster than Alp14-accelerated MTs grown from substrate-attached GMPCPP seeds (unfilled data points in Fig. 5A,B), suggesting that MTs nucleated by Alp14 have a larger complement of Alp14 at their plus ends which tends then to stay resident and drive faster subsequent growth. In the absence of Alp14, new MTs are not nucleated under these conditions. We suspect that small amounts of Alp14 are also present on the lattice of these fast-growing, Alp14-nucleated MTs, because they bind loosely to the Tween 20 passivated surface, whilst MTs formed at higher tubulin concentrations in the absence of Alp14 do not.

### Mathematical Model

**Figure 6:**
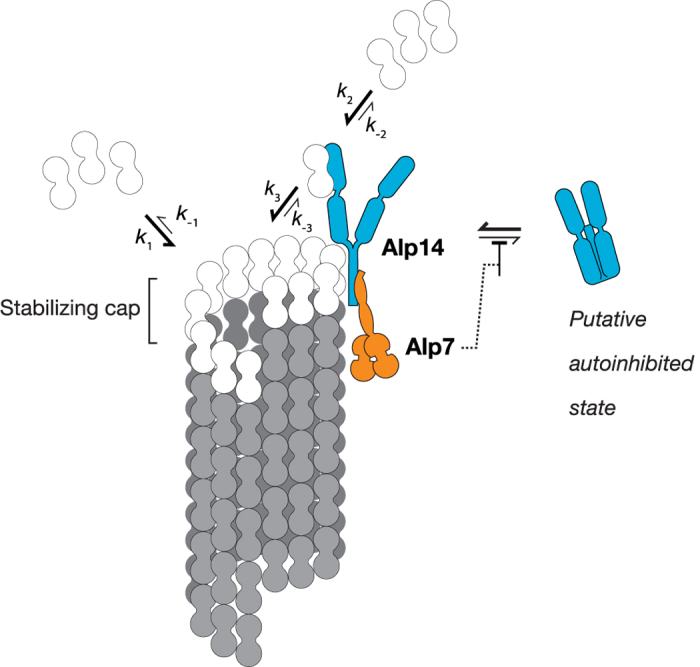
Model mechanism for Alp14-driven plus end growth Alp14 (blue) binds Alp7 (orange) and the complex tracks plus ends using a combination of TOG-based binding and C-terminal non-TOG binding. Tubulin in the terminal layer of the GTP-cap can either exchange directly with GTP-tubulin in solution (via *k*_1_) or indirectly via the TOGs of Alp14 (via *k*_2_ and *k*_3_). Alp7 binding to the C-terminal tail domain of Alp14 potentiates Alp14 residency at plus ends. One possibility is that Alp7 blocks the transit of Alp14 into a putative autoinhibited state, shown at right.

To build a quantitative model for the Alp14 mechanism (diagramed in Fig. 6 and discussed, with rationale, in Supplementary Material), we first assume the classical model for GTP-tubulin incorporation into MTs, a bimolecular reaction independent of Alp14 (justified by Fig. 3, shown in Fig. 6). We then introduce a competing facilitated pathway by which GTP tubulin can exchange into and out of the GTP cap of the MT via the TOG domains of Alp14. We assume that Alp14 tracks growing tips processively by diffusing rapidly on to newly-incorporated subunits (Fig. 4E, inset), and we further assume that lateral diffusion of Alp14 around the MT tip is rapid. Fast lateral diffusion of Alp14 between neighbouring PFs is required to allow single Alp14 molecules to accelerate growth uniformly across all PF tips in the MT, and multiple Alp14 molecules to accelerate growth independently (Fig. 4E). We fitted rate constants for this Alp14-facilitated pathway to reproduce the measured relationships between growth rate, Alp14 concentration and tubulin concentration (Figs 3 and 4; fitted values are summarized in Supplementary Table S1).

Of particular note, we find that our data cannot be fitted with a conventional model in which Alp14 accelerates the on- and off-rates of GTP-tubulin to an equal extent. Models of this type do not predict MT growth at sub-critical tubulin concentrations in the presence of Alp14; see Supplementary Material. Instead, our data indicate that Alp14 differentially changes the on- and off-rates, accelerating the on-rate of GTP-tubulin while stabilising the off-rate of GTP-tubulin.

A consequence of this unconventional mechanism is that Alp14 is predicted to influence the GTP cap at the MT tip, with implications for catastrophe. We avoid proposing a specific pathway for catastrophe. Instead, we posit simply that the catastrophe rate varies according to the time-averaged number of missing GTP-tubulin subunits in the stabilising terminal cap^25^. We call this the gap-number. In our model, Alp14 reduces the gap-number compared to tubulin alone at high tubulin concentrations, but increases the gap-number at low tubulin concentrations (see Fig. 5 and Supplementary Material).

In our model, the detailed coupling between gap number, GTP-tubulin cap depth and the probability of catastrophe is adjustable without affecting the overall behaviour. To explain the influence of Alp14 on catastrophe rate we require only that the probability of catastrophe depends monotonically on the gap number. Equipped with our quantitative model, we can now ask, how does Alp7 influence the assembler activity of Alp14 ? 

### Alp7-14 complexes generate very long MTs

In the presence of Alp14, Alp7 causes MTs to grow very much longer (Supplementary Movie 3). Mean MT length in 50 nM Alp14 was 3.6 μm whereas in 50 nM Alp14 plus 100 nM Alp7, mean MT length was 18.6 μm. This dramatic increase in mean MT length is due to a substantial suppression of catastrophe combined with a small increase in plus end growth rate (Table 2). In 50 nM Alp14, the MT plus end growth rate was 10.3 nm s^−1^ and the catastrophe frequency was 0.16 min^−1^. Adding 100 nM Alp7 increased the mean growth rate to 15.2 nm s^−1^ and decreased the catastrophe frequency to 0.04 min^−1^. We conclude that Alp7 strongly potentiates the assembler activity of Alp14 by increasing its occupancy, thereby increasing the plus end growth rate and reducing the plus end catastrophe rate so as to produce very long microtubules.

### Alp7 potentiates tip-tracking by Alp14

**Figure 7:**
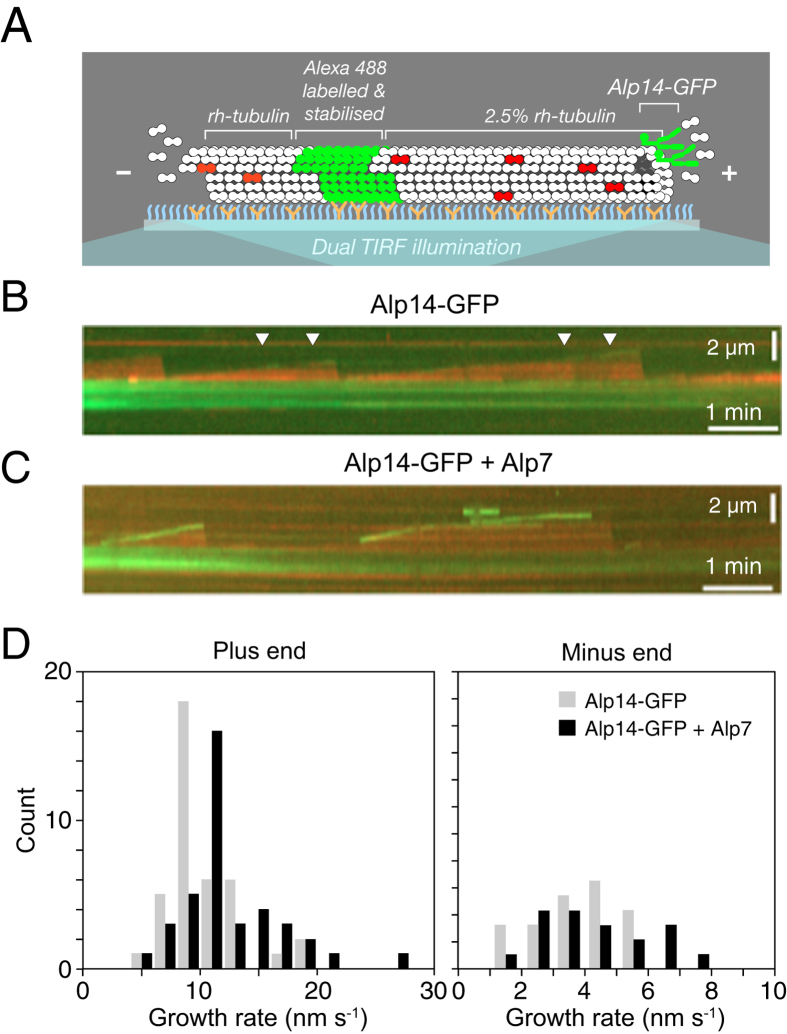
Alp7 potentiates tip tracking by Alp14 (**A**) Schematic of dual-TIRF assay. MTs are nucleated by immobilized Alexa488 labeled seeds using 6 μM *S. pombe* tubulin “doped” with 2.5% rhodamine labeled brain tubulin. (**B**) Alp14-GFP is introduced and a weak tip tracking signal is visible (white arrows). (**C**) Adding Alp7 enhances tip tracking by Alp14-GFP. (**D**) Adding Alp7 also significantly increases the mean plus end growth rate and decreases the catastrophe frequency, producing long-lived, fast growing MTs (Supplementary Movie 3) with no significant effect on minus ends (Table 2).

Al Bassam *et al.*^3^ recently assayed the effects of Alp14 on brain MTs, and reported complex effects, with inhibition under some conditions. Under our conditions, adding 5% brain tubulin to an Alp14-GFP-catalysed *S. pombe* MT polymerisation mix fully competitively inhibits the assembler activity of Alp14. MTs remain dynamic, but rates of growth, shrinkage and rescue revert to those for pure *S. pombe* tubulin. With 6 μM *S. pombe* tubulin and 2.5% (150 nM) rhodamine brain tubulin (Fig. 7), tip tracking by Alp14 is heavily depleted, such that a weak Alp14-GFP signal appears only intermittently at MT plus ends (white arrows in Fig. 7B; Supplementary Movie 4) and plus end growth is only slightly elevated, to 10.2 nm s^−1^. To visualize directly the effect of Alp7 on Alp14 occupancy, we set up a TIRF experiment in which brain tubulin is used to reduce tip tracking by Alp14-GFP on *S. pombe* MTs. Addition of Alp7 under these conditions rescues tip tracking by Alp14-GFP (Fig. 7C; Supplementary Movie 4), but mean plus end growth increases only marginally, to 12.4 nm s^−1^ (Fig. 7D); Nonetheless, catastrophe is suppressed, consistent with our core proposal that Alp14 stabilises the GTP-cap via TOG-independent binding. We conclude (1) that in the absence of Alp7, tip tracking by Alp14 depends heavily on active catalysis of tubulin exchange by the TOG domains and (2) that Alp7 can restore tip tracking by Alp14 under conditions where Alp14 is otherwise diminished. Notably, even though Alp7 restores tip tracking by Alp14 and growth episodes last longer (Supplementary Movie 4), growth rate is little affected. This is consistent with our model, because under these conditions, whilst tip-stabilisation by the non-TOG binding of Alp14 is expected, accelerated growth is not, since the tip tracking Alp7-14 complexes are still competitively inhibited by brain tubulin.

## Discussion

### Alp14 differentially accelerates the on- and off-rates for GTP-tubulin at plus ends

Our data reveal that Alp14 not only accelerates the exchange of GTP-tubulin at plus ends, but also biases the equilibrium constant for GTP-tubulin exchange, by substantially increasing the on-rate whilst having little or no overall effect on the off-rate. This is not the behavior of a classical enzyme, since enzymes do not shift equilibrium constants. What causes the anomalous behavior of Alp14 ? In our model (Fig. 6), Alp14 combines conventional catalysis of GTP-tubulin exchange by its TOG domains with tip-lattice stabilisation by a non-TOG binding site in the Alp14 tail. This combination of catalysis of tubulin exchange with stabilisation of the GTP-tubulin tip lattice means that as more Alp14 is loaded, the faster the MT plus end tip grows, and the more stable it gets. There is strong evidence that the C-terminal tail of Alp14 binds to MTs independently of the TOG domains^3^. Other TOG family polymerases, for example XMAP215, also make non-catalytic binding interactions with the MT lattice^32^, but there is no evidence that this stabilises the tip-lattice significantly, since XMAP215 has been shown not to bias the GTP-tubulin exchange equilibria^21^. The tip-stabilising ability of Alp14 is expected to be especially effective for *S. pombe* MTs, since as we have shown, these have a 5-fold lower basal GTP-tubulin off-rate than brain MTs, combined with a similar second order GTP-tubulin on-rate. This reduces the critical GTP-tubulin concentration for plus end growth of *S. pombe* MTs by about 5-fold compared to brain MTs, and means that Alp14’s non-TOG stabilisation of the GTP-cap of *S. pombe* MTs is acting to reduce further an already very low basal GTP-tubulin off-rate.

### The effects of Alp14 on catastrophe depend on occupancy and the free GTP-tubulin concentration

Whilst the unusual mechanism of Alp14 allows it to drive MT growth at very low GTP-tubulin concentrations, it does so at the expense of increased catastrophe. Our model predicts that at low GTP-tubulin concentrations, many Alp14 molecules must load so as to maintain the plus end growth rate. Because of the low free tubulin concentration, each of these tip-resident Alp14 molecules will spend appreciable amounts of time with its catalytic sites (its TOG domains) empty, waiting for free GTP-tubulin to land. This increases the probability that the TOGs will extract GTP-tubulin from the cap, so increasing the gap number and favouring catastrophe. As a result, at low tubulin concentrations and high Alp14 concentrations, microtubules grow fast but have a high catastrophe rate. By contrast, at high tubulin concentrations, fewer tip-resident Alp14 molecules are required and those that are resident will spend most of their time capturing GTP-tubulin from solution and inserting it into the cap. At higher tubulin concentrations therefore, Alp14 suppresses catastrophes, causing MTs to grow both faster and longer.

### Alp7 potentiates the plus end polymerase activity of Alp14 by increasing its occupancy

Alp14 accelerates the on-rate for GTP-tubulin at MT plus ends and Alp7 binds tightly to the C-terminal domain of Alp14. We find that Alp7 increases the occupancy of Alp14 (Fig. 7C), thereby potentiating its ability to accelerate plus end growth. Alp7 is required for the full polymerase activity of Alp14 *in vivo*, revealing that Alp7 binding serves not only to direct Alp14 to specific mitotic targets, but also more generally to potentiate Alp14 polymerase activity throughout the cell cycle, by enhancing its occupancy at MT plus ends. We speculate that like Stu2^17^, Alp14 may tend to fold up into an auto-inhibited conformation (‘Putative autoinhibited state’ in Fig. 6), and that Alp7 binding might disfavour Alp14 auto-inhibition.

### Comparing the Alp14 mechanism to that of other TOG family polymerases

For Stu2, it was recently shown that a linked pair of TOGs (TOGs 1 and 2) can bind two tubulins^29^. Based on this, Ayaz and colleagues propose that one TOG of each chain serves to recognise and track the MT tip whilst the other recruits GTP-tubulin from solution^29^. Our own results refer exclusively to full length Alp14. Our model predicts the flux across each tip-bound Alp14 molecule at a particular free tubulin concentration, but this refers to the collective properties of the 4 TOGs – at present we have no information about their individual contributions. It is clear that competitive inhibition of the TOGs of Alp14 by brain tubulin reduces tip-tracking (Fig. 7), consistent with a role for the TOG domains in tip-tracking. However, we also find evidence that Alp14 binds the GTP-cap via an additional non-TOG C-terminal site, and that this stabilises the cap. Binding of Alp14 to the GTP-cap via this additional non-TOG site might allow all four TOGs to detach transiently without triggering dissociation of Alp14, potentially increasing processivity and allowing the TOGs of Alp14 to act more independently. Our model refers to intact Cut7 tetramers, and we cannot currently infer the roles of individual TOG domains. Further work will be required to explore these points.

### The Alp7/TACC-Alp14/TOG mechanism is tuned to *S. pombe* MTs

Our finding that brain tubulin can competitively inhibit Alp14-driven growth of *S. pombe* MTs emphasises that for TOG-family polymerases to be effective, the TOG domains need to be functionally matched to their tubulin substrate, so as to tune the requirement for tight and specific binding of GTP-tubulin from solution with the potentially conflicting requirement to donate GTP-tubulin rapidly into the tip-lattice. With *S. pombe* MTs, Alp14 both accelerates plus end growth and lowers the already-low critical GTP-tubulin concentration for plus end growth. Mismatching of tubulins and TOGs risks producing a too-stable TOG-tubulin complex and thereby quenching polymerase activity. As noted above, Podolski and colleagues^22^ recently reported that Stu2 is a much better polymerase for *S. cerevisae* tubulin than it is for brain tubulin. We find that whilst brain tubulin competitively inhibits the action of Alp14 on dynamic *S. pombe* MTs, Alp14 nonetheless does accelerate the shrinkage of shrinking GMPCPP brain MTs (not shown). This is consistent with our model, because diluted GMPCPP brain MTs shrink slowly enough ( < 1 nm s^−1^) that the off-rate of brain tubulin from Alp14 is nonetheless fast enough to accelerate their shrinkage.

### General implications for TOG-polymerase driven growth of MTs in cells

*In vitro*, Alp7/14 promotes MT nucleation as well as MT growth. *In vivo*, deletion of Alp7/14 significantly reduces the number of IMAs per cell. This argues that Alp7/14 *in vivo* may drive MT nucleation, and then track and accelerate the growth of MT plus ends as they extend away from the nucleation site. Further work will be required to explore this possibility. In living *S. pombe*, the total tubulin concentration is estimated at 1-5 μM (Ref3 and Drummond & Cross, unpublished). As we have shown here, the intrinsic GTP-tubulin off-rate at the plus ends of *S. pombe* MTs is about 5-fold lower than for brain MTs, which explains how the relatively low *in vivo* tubulin concentration in *S. pombe* can support the fast and elaborate MT dynamics seen in live cell imaging. Even taking our upper bound of 5 μM total tubulin *in vivo*, and assuming this is all in the form of free GTP-tubulin (which it clearly is not), our measured rate constants predict that MTs built from dual isoform *S. pombe* tubulin would still only grow at ~14 nm s^−1^ (Fig. 3) whereas the measured plus end growth rate of interphase MTs in living *S. pombe* is ~50 nm s^−1^ 30. Based on our measurements, driving MTs to grow at the *in vivo* rate of 50 nm s^−1^ will require ~10 molecules of Alp14 per plus end. We conclude that the accelerate-and -bias catalytic mechanism of Alp14, the occupancy-enhancing action of Alp7 and the unusually low basal GTP-tubulin off-rate of *S. pombe* MTs are all necessary in order to achieve the fast and sustained MT growth seen at cellular tubulin concentrations in living *S. pombe*.

## Methods

### Yeast cell biology

*S. pombe* strains listed in Supplementary Table S2 were a kind gift from Takashi Toda (Cancer Research UK, London Research Institute). Strains had been derived from those in^6^ by the insertion of GFP tubulin fusions. *S. pombe* cells were cultured using standard methods^33^. For imaging, cells were grown in starter cultures of YES media at 25 °C then diluted into EMMG, 100 μg ml^−1^ uridine, 100 μg ml^−1^ leucine and grown with shaking at 25 °C to early log phase ~2 × 10^6^ cells ml^−1^. Doubling times of each of the populations were ~9 hours for *alp7* or *alp14* deletions and ~5.5 hours for control cells.

### Time-lapse live cell imaging

Aliquots of cells in early log phase were placed on a pad of agar (2% (w/v) agarose in EMMG, 100 μg ml^−1^ uridine, 100 μg ml^−1^ leucine) on a microscope slide. A coverslip was placed on top and sealed with VALAP (Vaseline, Lanolin, Paraffin wax in a 1:1:1 mix). Slides were incubated at 25 °C or 35 °C for > 1 hour prior to imaging. A heated microscope stage was used to maintain the temperature during imaging using a spinning disk confocal microscope: PerkinElmer UltraVIEW VoX confocal imaging system with a Yokagawa CSU-X1 disk unit, Hamamatsu ORCA-R2 camera and a Nikon ECLIPSE Ti microscope controlled by PerkinElmer Volocity v 6.1 software. Images were captured at 0.1 fps using a 100 × 1.4NA objective and 2x camera binning, giving an equivalent pixel size of 139 nm.

### Cloning of Alp14 and Alp7

The genes encoding Alp14, Dis1 and Alp7 were PCR-amplified from wildtype genomic *S. pombe* DNA. DNA encoding Alp14 with a C-terminal 10× Histidine tag was cloned into the pIEx/Bac-NcoI = > NdeI via NdeI and NotI. Alp14 with a C-terminal AviTag was cloned into the pIEx/Bac-C-Avi via NdeI and NotI. Alp14 with an N-terminal AviTag was cloned into the pIEx/Bac-N-Avi via NdeI and NotI. Alp7 with a N-terminal AviTag was cloned into the pIEx/Bac-N-Avi via NdeI and NotI. The gene of the BirA-Ligase was amplified from a pET-17b vector and cloned into the pIEx/Bac-NcoI = > NdeI vector via NdeI and SacI, adding a stop-codon in front of the His-tag.

### Protein expression in Sf9-insect cells, using the baculovirus BacMagic system (Novagen)

Proteins were expressed in a baculovirus system, using insect Sf9 cells. Sf9 cells were incubated at 28 °C with shaking at 100–200 rpm for virus propagation and protein expression.

### Creating baculovirus from the pIEx/Bac-plasmids

pIEx/Bac vectors were propagated over night in a 100 ml DH5 bacterial cell culture at 37 °C and purified with the EndoFree Plasmid Maxi Kit (Qiagen).

### Expression and solubility test before baculovirus generation

Expression and solubility of proteins were checked using the InsectDirect system from Novagen, which allows the translation of the proteins directly from the pIEx/Bac-vector, following the manufacturer’s instructions. After 48 h Sf9-cells were collected and lysed as described below for larger Sf9 cultures. The lysate was spun at 20,000 g. Pellet and supernatant were tested by Western Blotting for the expressed proteins.

### 1st baculovirus generation

The first generation of baculovirus was generated using the BacMagic system from Novagen. According to the manufacturer’s protocol, a mixture of 1 ml Bac Vector Insect Cell Media (Novagen), 5 μl Insect GeneJuice Transfection Reagent (Novagen), 500 ng pIEx/Bac-vector and 100 ng BacMagic DNA (Novagen) was incubated for 15–30 min at room temperature. The mixture was then added to 1 × 10^6^ cells in a 35 mm dish after removal of the initial Bac Vector Insect Cell Medium. Cells were incubated at 28 °C in a humidified container. After 5 h another 1 ml of Bac Vector Insect Cell Media was added to avoid drying. After 5 days the medium was harvested and used as the first generation of baculovirus.

### Baculovirus amplification

0.3 ml of 1st generation baculovirus (P1 virus) were added to 30 ml of Sf9 cells at 1.5 × 10^6^ cells ml^−1^ in Bac Vector Insect Cell Media, supplemented with 1.5% (v/v) Fetal Bovine Serum (FBS) (Sigma), 100 U/ml penicillin and 100 μg ml^−1^ streptomyocin (GIBCO/Invitrogen), shaking at 200 rpm at 28 °C for 3 to 5 days. When cells started to lyse and the cell number decreased, the supernatant containing P2 virus was harvested by centrifugation at 200 g for 5 min. For long term storage 1 ml aliquots were frozen by first incubating for 1 h at 20 °C, then for 24 h at −80 °C before storage in liquid nitrogen. Working stock solutions of P3 baculovirus for expression were made by a 2nd virus amplification. 0.3 ml of P2 virus was added to a 30 ml suspension culture of 1 × 10^6^ Sf9 cells ml^−1^. Cultures were incubated at 28 °C at 200 rpm for 3–5 days. When cells started to lyse, the supernatant was collected after centrifugation at 200 g and used as P3 virus for infection of larger Sf9 cell cultures.

5 ml of P3 virus was added to 500 ml of 1 × 10^6^ Sf9 cells ml^−1^ and incubated for 54–64 h at 28 °C, shaking at 120 rpm. For co-expression of BirA-Ligase and Avi-tagged proteins, 4 ml of P3 virus of the target protein and 1 ml of BirA-Ligase P3 virus were added, to ensure biotinylation of the Avi-tag. Cells were harvested by centrifugation at 100 g. The wet cell pellet was weighed and subsequently lysed in lysis buffer (50 mM HEPES buffer pH 7.5, 150 mM NaCl, 5% (v/v) glycerol, 0.1% (v/v) Triton-X 100, supplemented freshly with 1 mM DTT (Sigma) and protease inhibitors: 5 μg ml^−1^ chymostatin (Peptide International Inc., USA/Canada), 0.5 μg ml^−1^ leupeptine (Peptide International Inc., USA/Canada), 10 μg ml^−1^ antipain (Peptide International Inc., USA/Canada), 2 μg ml^−1^ aprotinin (Peptide International Inc., USA/Canada), 0.7 μg ml^−1^ pepstatin (Peptide International Inc., USA/Canada), 10 μM E64 (Peptide International Inc., USA/Canada), 10 μg ml^−1^ PMSF (Sigma)), using 4 ml lysis buffer per 1 g wet pellet. Cell lysates were quick frozen by plunging into liquid nitrogen. The lysate was then used for further purification or was stored at −80 °C.

### Protein purification

Because proteins were expressed with different affinity tags, the purification procedure for each construct varied. Frozen lysates was thawed on ice and centrifuged at 20,000 g for 40 min at 4 °C in an SS-34 rotor (Sorvall). Supernatants were then further processed at 4 °C. Alp14 and Alp7 and different affinity tags required different conditions for successful purification.

### Purification of Alp14 with a His-tag

For purification of His-tagged proteins without a GFP-label, 50 ml clarified lysis supernatant was loaded at 5 ml min^−1^ on to a 5 ml HiTrap SP HP column (GE Healthcare), equilibrated with a low-salt cation exchange buffer (150 mM NaCl, 6.7 mM HEPES pH 7.5, 6.7 mM MES, 6.7 mM Na-Acetate). The column was sequentially washed with ~25 ml low-salt cation exchange buffer and then ~25 ml cation exchange buffer containing 250 mM NaCl. Elution used a continuous gradient from 150 mM NaCl to 1000 mM NaCl in the cation exchange buffer. Alp14 started to elute from 300 mM NaCl onwards. Aliquots were checked by SDS-PAGE, peak fractions pooled, imidazole added to 10 mM, then bound to a 1 ml HisTrap HP column, pre-equilibrated with low-imidazole buffer (50 mM NaPO_4_ pH 8.0, 300 mM NaCl, 15 mM imidazole, 10% glycerol). The column was sequentially washed with imidazole buffer (50 mM NaPO_4_ pH 8.0, 100 mM NaCl, imidazole, 10% glycerol) containing 30, 60, 80 mM imidazole and then eluted with buffer containing 500 mM imidazole. Alp14 fractions were pooled and loaded onto a 1 ml HiTrap Q HP column (GE Healthcare) equilibrated with 10 mM BisTris propane pH 8.0, 10 mM Tris-HCl, 10% glycerol, then eluted using a 100−1000 mM NaCl gradient. Alp14 containing fractions were pooled, aliquoted into 3 - 4 20 μl aliquots containing ~4 μM Alp14 and quick-frozen in liquid nitrogen.

### Purification of Alp14-GFP

For purification of Alp14-GFP, 10–20 ml of cleared lysate was loaded on to a 1.2 ml SP Sepharose HP (GE Healthcare) column equilibrated with low-salt cation exchange buffer. The resin was washed with 7.5 ml low-salt cation exchange buffer and 10 ml of 250 mM NaCl in cation exchange buffer. Proteins were eluted with 10 ml of 500 mM NaCl in cation exchange buffer. Fractions containing the target protein were pooled and loaded onto 500 μl of Ni-NTA Superflow resin (Qiagen) equilibrated with low-imidazole buffer. The resin was washed with 10 ml of low-imidazole buffer then imidazole buffer containing 30 and 60 mM imidazole buffer before elution with 500 mM imidazole buffer. Peak fractions containing Alp14 were pooled, aliquoted, quick frozen and stored in liquid nitrogen.

### Purification of AviTag proteins

Avi-tagged proteins were purified by applying 15–20 ml lysate supernatant to a 600–800 μl Monomeric Avidin Agarose (Thermo Scientific) gravity flow column equilibrated in 100 mM NaPO_4_ pH 7.2 and 150 mM NaCl. The resin was washed with 10 ml of (100 mM NaPO_4_ pH 7.2 and 150 mM NaCl), and 10 ml of (100 mM NaPO_4_ pH 7.2 and 450 mM NaCl). Without the second wash at 450 mM NaCl concentrations, insect cell tubulin co-purified with Alp14. Proteins were first eluted with 10 ml elution buffer (100 mM NaPO_4_ pH 7.2, 150 mM NaCl and 7.5 mM biotin) then with 3 ml elution buffer containing 2 M urea and 500 μl fractions collected. Urea-free peak fractions were supplemented with 10% glycerol and 20 or 50 μl aliquots frozen and stored in liquid nitrogen. Peak fractions containing 2 M urea were dialysed against 100 mM NaPO_4_ pH 7.2, 150 mM NaCl and 10% glycerol in a Slide-A-Lyzer Dialysis Cassette 7 K MWCO (ThermoScientific) according to the manufacturers instructions. The dialysed proteins were then aliquoted, quick frozen and stored in liquid nitrogen.

### Purification of tubulins

*S. pombe* tubulins were prepared according to^34^ from wild type strains containing dual isoform α1β + α2β or from a single isoform strain (mmsp174) expressing α1β tubulin^35^. Pig brain tubulin was prepared as described in^36^, desalted into PEM plus 20 μM GDP and stored in liquid nitrogen. Tubulin concentrations were measured in 6 M GdHCl using extinction coefficients of 105 838 M^−1^ cm^−1^ (pig brain) and 108 390 M^−1^ cm^−1^ (*S. pombe*) calculated according to^37^. Polymerisation-competent tubulin was estimated by polymerising tubulin at 25 °C for 90 min in PEM, 1 mM DTT, 1 mM GMPCPP. MTs were pelleted in a TLA100 rotor (Beckman Coulter) at 45,000 rpm (90,000 g), 25 °C for 5 min. Samples of SN and pellet were analysed by SDS-PAGE and Sypro Red staining (Invitrogen). The proportion of functional polymerized tubulin in the pellet was determined using a Pharos laser gel scanner and Quantity one software (Bio-Rad) as 86.6% of single isoform and 88.9% of wild type tubulin.

### Microtubule dynamics assays

GMPCPP-stabilised pig brain MT seeds labelled with Alexa488 were created using 5 μM pig brain tubulin (10% labelled with Alexa Fluor 488 succinimidyl ester (A-20000, Molecular Probes)) in 1 mM GMPCPP, 1 mM MgCl_2_, PEM100, incubated on ice for 5 min to exchange the GTP and GMPCPP then polymerised at 37 °C for 45 min. Microtubules were centrifuged in an Airfuge (Beckman Coulter) for 5 min at 25 psi at room temperature and the microtubule pellet was carefully resuspended in 50 μl warm PEM100. Flow chambers were prepared using ~3 mm wide strips of double stick tape, spaced ~6 mm apart on a silanized slide glass. Attaching a silanized cover glass (22 mm × 22 mm) formed 3 channels. All standard solutions such as PEM100-buffer, glucose oxidase, catalase and glucose were clarified by centrifugation in a TLA 100.3 rotor (Beckman Coulter) at 50 000 × g for 30 min.

Alp14 and Alp7 were buffer exchanged into PEM-buffer via spin columns (ZebaSpin Desalting Columns, Micro (75 μl), 40 K MWCO) before each set of experiments. Concentrations of Alp14 and Alp7 in the buffer-exchanged and centrifuged samples were measured after the experiments by densitometry of SyproRed (Cytoskeleton Inc) stained SDS-PAGE gels. A mastermix was prepared containing 1 mM GTP, 38 U ml^−1^ glucose oxidase, 8 ng ml^−1^ catalase, 1% (v/v) β-mercaptoethanol, 4.5 mg ml^−1^ glucose in PEM100-buffer. Solutions, including rebuffered Alp14, Alp7, reaction mastermix and diluted Anti-Alexa Fluor 488 antibody (A-11094, Molecular Probes) were cleared by centrifugation for 5 min at 20 800 g at 4 °C immediately before each experiment. The reaction mix was prepared by adding 3 μl of *S. pombe* tubulin and 3–6 μl of buffer exchanged protein to the mastermix, plus buffer to a total volume of 20 μl. Flow chambers were conditioned as follows. Using a vacuum pump and subsequently Whatman paper (Grade 1, Whatman), 50 μl PBS was flushed through the channel to hydrate the channel. 15 μl of 0.1 mg ml^−1^ Anti-Alexa Fluor 488 antibody (A-11094, Molecular Probes) in PBS-buffer was flowed into the chamber and incubated for 5 min. The channel was washed with 40 μl PEM100- buffer. The surface was then blocked with 20 μl 1% (v/v) Tween-20 for 30 min. The channel was then washed with 80 μl PEM100. 15–20 μl 1:200 diluted GMP-CPP stabilised microtubule-seeds labeled with Alexa488 were flushed into the channel and incubated for 5 min. The channel was washed again with 40 μl PEM100. Finally 20 μl freshly prepared reaction mix was flushed into the channel. The channel was sealed with VALAP (Vaseline, Lanolin, Paraffin wax in a 1:1:1 mix) and imaging begun within 20 min.

### Dark-field microscopy

Microtubule dynamics were imaged as described in^38^ using a Nikon E800 microscope fitted with a Plan Fluor 100×/0.5–1.3 oil iris (Nikon) and a dark- field oil condensor (Nikon). Dark-field illumination of dynamic unlabeled *S. pombe* microtubules was generated by a mercury arc lamp (HBO100/W2, Zeiss) using a fibre optic light scrambler (Technical Video Ltd.) to ensure even illumination of the condensor. To protect the sample from light damage, including UV-light, a green interference filter (Nikon) was used. In epifluorescence, the Alexa Fluor 488 seeds were excited by a mercury arc lamp (X-Cite exacte system, Lumen Dynamics) via an excitation filter HQ470/40× (Semrock) and a dichroic mirror Q495LP (Chroma Technology). Imaging used an emission filter HQ525/50 m (Semrock). Shutters in front of the light sources enabled switching from dark-field to epifluorescence, controlled by a MAC5000 controller (LUDL electronic products Ltd.). Dark-field images were taken every 1 s with an exposure time of 100 ms while epifluorescence images were taken every 60 s with an exposure time of 200 ms. Videos were taken for a duration of 10–20 min. Images were captured with an Andor iXON + DU-897E EMCCD camera (Andor Technology), cooled to −80 °C. The system was controlled using Metamorph imaging software (Molecular Devices Inc). To maintain the microscope at 25 °C, an insulated box around the microscope was heated by an AirTherm ATX system (World Precision Instrument, Inc.)

### DIC microscopy and assay of unlabeled MT dynamics

MT dynamics assays^27^ of unlabeled *S. pombe* wild type and single isoform tubulins using VE-DIC microscopy to image MTs nucleated by axoneme fragments were carried out and analysed as described in^38^ except that assays were in PEM buffer, 1 mM DTT, 1 mM GTP at 25 °C. Plots of MT length against time were analysed manually to identify phases of MT growth and shrinkage and the rates determined by linear regression (Kaleidagraph, Synergy software). Phases with length changes < 0.24 μm are classified as pauses. Catastrophes are the number of catastrophe events divided by total growth time. MT growth rates were plotted against tubulin concentration after correcting for non-functional tubulin.

### Data analysis

Analysis of fluorescence and darkfield data used Metamorph (Molecular Devices Inc.). MT tips were tracked with the custom macro GetEdge for ImageJ (http://mechanochemistry.org/Cross), written by Miho Katsuki. Microtubule dynamics were analysed by selecting a microtubule of interest, converting it into a kymograph and measuring its length change over time. Statistical analysis used Prism software.

## Additional Information

**How to cite this article**: Hussmann, F. *et al.* Alp7/TACC-Alp14/TOG generates long-lived, fast-growing MTs by an unconventional mechanism. *Sci. Rep.* **6**, 20653; doi: 10.1038/srep20653 (2016).

## Supplementary Material

Supplementary Information

Supplementary Video 1

Supplementary Video 2

Supplementary Video 3

Supplementary Video 4

## Figures and Tables

**Table 1:** *S. pombe* tubulin MT polymerisation kinetics MT growth rates were plotted against GTP tubulin concentration for *S. pombe* single isoform (α1β) and wild type (α1β, α2β) tubulin at 25 °C.

Tubulin isoforms	plus end			minus end		
	k_on_ nm s^−1^ μM^−1^	k_off_ nm s^−1^	k_off_/k_on_ μM	k_on_ nm s^−1^ μM^−1^	k_off_ nm s^−1^	k_off_/k_on_ μM
α1β	3.4	4.1	1.2	1.9	1.7	0.9
α1β, α2β	4.7	9.3	2.0	1.6	3.1	1.9

Apparent rate constants for MT polymerisation were determined by linear regression using a simple polymerisation mechanism where: Growth rate per MT end = k_on_ [tubulin] − k_off_ Units in nm of MT growth are used as the protofilament number is unknown. Assuming 13 or 14 protofilaments conversion factors of 1.62 or 1.75 will convert the values from nm to heterodimers. The critical concentration for MT polymerisation is given by the calculated K_d_ = k_off_/k_on_. The critical concentration is similar at the plus and minus ends in both single and double isoform *S. pombe* tubulin whilst the rate constants are faster at the plus end.

**Table 2:** Microtubule growth rate, mean length & catastrophe frequency in Alp14 and Alp7 Alp7 greatly enhances the action of Alp14, producing a further increase in growth rate and a substantial increase in MT length together with reduced catastrophe frequency at MT plus ends.

	Plus end growth nm s^−1^	Plus end catastrophe frequency min^−1^	MT length μm	Minus end growth nm s^−1^	Minus end catastrophe frequency min^−1^
50 nM Alp14	10.3 ± 3.2 (39)	0.16	3.6 ± 4.4 (24)	2.6 ± 1.6 (27)	0.15
80 nM Alp14	13.4 ± 4.0 (38)	0.12	6.5 ± 5.7 (29)	2.3 ± 1.0 (19)	0.1
50 nM Alp14 + 100 nM Alp7	15.2 ± 4.2 (9)	0.04	18.6 ± 12.4 (8)	2.5 ± 1.3 (11)	0.15

Mean values ± SD (n). Mean growth rates at plus ends are significantly different (ANOVA, p = 0.0001). The growth rate of either 80 nM Alp14 alone or 50 nM Alp14 + 100 nM Alp7 were both significantly faster than 50 nM Alp14 alone. There was no significant difference between the growth rate of 80 nM Alp14 alone or 50 nM Alp14 + 100 nM Alp7 (Tukey post test, p < 0.01). There was no significant difference between the mean growth rates at MT minus ends (ANOVA, p = 0.7656). The mean MT lengths were significantly different (ANOVA, p < 0.0001). 50 nM Alp14 + 100 nM Alp7 was significantly longer than either 80 nM or 50 nM Alp14 alone. There was no significant difference between 50 and 80 nM Alp14 (Tukey post test p < 0.001).
