# Supplementary material for: Alp7/TACC-Alp14/TOG generates long-lived, fast-growing MTs by an unconventional mechanism
Source: Sci Rep. 2016 Feb 11;6:20653. doi: 10.1038/srep20653 (PMC4749977; doi:10.1038/srep20653)
Supplement: Supplementary Information [file srep20653-s1.pdf]

# Alp7/TACC-Alp14/TOG generates long-lived, fast-growing MTs by an unconventional mechanism

Frauke Hussmann, Douglas R. Drummond, Daniel Peet, Douglas S. Martin & Robert A. Cross*

Warwick Medical School, Coventry CV4 7AL UK

*Corresponding author: r.a.cross@warwick.ac.uk

## SUPPLEMENTARY INFORMATION

**A simple Alp14 model**

The following model attempts to account for *S. pombe* microtubule dynamics in the absence and presence of Alp14. The constraints on the model are the following:

- Microtubule growth in the absence of Alp14 (Figure 3 in main text)
- Average microtubule growth in the presence of Alp14 (Figure 4 in main text)
- Maximum microtubule growth with Alp14 and 6 $\mu$M tubulin is ~35 nm $s^{-1}$, independent of Alp14 concentration (including for *de novo* nucleated MTs)
- Growth at 0.5 $\mu$M tubulin of 12 nm $s^{-1}$ in the presence of Alp14 (Figure 5)
- At least one Alp14 per MT plus end in the Alp14 concentration ranges of the experiments

| ***Rate*** | ***Description*** | ***Value*** | ***Comment*** |
|---|---|---|---|
| $k_1$ | tubulin on-rate w/o Alp14 | 5.5 $\mu M^{-1} s^{-1}$ | Fit of eq. 1 to data in Fig. 3A; brain tubulin 8.9 $\mu M^{-1} s^{-1}$ (**walker 1988**) |
| $k_{-1}$ | tubulin off-rate w/o Alp14 | 6.6 $s^{-1}$ | Fit of eq. 1 to data in Fig. 3A; brain tubulin 43 $s^{-1}$ (**walker 1988**) |
| $K_c$ | critical concentration for tubulin growth | 1.2 $\mu$M | $k_{-1}/k_1$; brain tubulin 5 $\mu$M (**walker 1988**) |
| $K_{c,Alp14}$ | critical concentration for tubulin growth with Alp14 | 0.08 $\mu$M | $(k_{-2}/k_2)(k_{-3}/k_3)$, fit of eq. 2 to data in Fig. 4C (see text for more) |
| $k_2$ | tubulin binding rate to Alp14 | >40 $\mu M^{-1} s^{-1}$ | required for 12 nm $s^{-1}$ growth at 0.5 $\mu$M tubulin |
| $k_{-2}$ | tubulin off-rate from Alp14 | 1-15 $s^{-1}$ | underconstrained (see text) |
| $k_3$ | tubulin on-rate via Alp14 | 6 $s^{-1}$ | constrained by data in Fig. 4C |
| $k_{-3}$ | tubulin off-rate via Alp14 | 15-1 $s^{-1}$ | underconstrained (see text) |
| $K_{D,Alp14}$ | dissociation constant for Alp14 from MT plus end | 6 nM | constrained by data in Fig. 4C and 5A (see text) |

**Table S1**. Rate constants for tubulin exchange at MT plus ends.

Microtubule polymerisation in the absence of Alp14 is modeled with a simple bimolecular reaction [1],

$$T + M \underset{k_{-1}}{\overset{k_1}{\rightleftharpoons}} (M + 1) \qquad (1)$$

where $T$ is a (GTP) tubulin dimer, $M$ is the microtubule plus end, and $(M + 1)$ is the microtubule plus end with an additional tubulin ligated (see Figure 6 left, main text). The forward rate ($k_1$) and backward rate ($k_{-1}$) are fit to microtubule growth of single isoform *S. pombe* tubulin in PEM at 25°C in the absence of Alp14, with $k_1 = 5.5\ \mu M^{-1} s^{-1}$ and $k_{-1} = 6.6\ s^{-1}$ (see Figure 3A, main text).

With Alp14 present, a second, Alp14-facilitated pathway is added to the model (Figure 6 right, main text),

$$T + M + E \underset{k_{-2}}{\overset{k_2}{\rightleftharpoons}} TE + M \underset{k_{-3}}{\overset{k_3}{\rightleftharpoons}} E + (M+1) \qquad (2)$$

where *E* is an Alp14 enzyme bound to the plus end of the microtubule and *TE* is a 1:1 tubulin-Alp14 complex bound to the plus end of the microtubule. The version of this model which we use has Alp14 able to move between protofilaments during growth, effectively averaging its effect across the entire MT plus end. We view this as justified by the data in Figure 4E: increasing Alp14 resident at plus ends results in higher growth rates. A fit using this model is shown in Figure 4, main text.

In principle, an even simpler Alp-14 facilitated pathway could combine the rates $k_{\pm 2}$ and $k_{\pm 3}$ into single accelerated on- and off-rates, given by

$$T + M \underset{k_{off}}{\overset{k_{on}}{\rightleftharpoons}} (M+1) \qquad (2alt)$$

This simpler pathway must be excluded due to the following experimental observation (Figs. 4,5 main text): At 0.5 $\mu$M tubulin, Alp14 microtubules grow at 12 nm s$^{-1}$ (20 dimers s$^{-1}$), implying an on rate of at least 40 $\mu$M$^{-1}$ s$^{-1}$ (higher when $k_{off}$ is included). At 6 $\mu$M, eq. 2alt would then predict at least 140 nm s$^{-1}$, much faster than the observed maximum of 35 nm s$^{-1}$ and average of 10-20 nm s$^{-1}$. Hence, we must include the more complicated pathway (eq. 2), and would expect fast Alp14-tubulin complex formation (a fast $k_2$) followed by a slower incorporation of tubulin into the microtubule ($k_3$), which becomes rate-limiting at higher tubulin concentrations to explain the observed maximum.

This model (Figure 6, main text, and equations 1 and 2) generates a set of five coupled differential equations, one each for *T, M,* (*M*+1), *E,* and *TE* as defined in (1) and (2) above. To solve, we take the perspective of a single microtubule plus end and assume steady-state: the growth rate is constant, the fraction of Alp14 occupied with tubulin and free from tubulin are constant, and the concentration of free tubulin remains constant. The five differential equations then simplify into a system of three linear equations:

$$-E(Tk_2 + k_{-3}) + TE(k_3 + k_{-2}) + \left[0\frac{d(M+1)}{dt}\right] = 0 = \frac{dE}{dt} \qquad \text{(unoccupied enzyme)}$$
$$-E(k_{-3}) + TE(k_3) - \frac{d(M+1)}{dt} = k_{-1} - Tk_1 \qquad \text{(microtubule growth)}$$
$$E + TE + \left[0\frac{d(M+1)}{dt}\right] = (E_{TOT}) \qquad \text{(total plus-end bound enzyme)}$$

These three linear equations are then solved for unoccupied enzyme *E*, occupied enzyme *TE*, and microtubule growth rate *d*(*M*+1)/*dt* via matrix inversion:

$$\frac{d(M+1)}{dt} = E_{tot}\frac{(Tk_2k_3 - k_{-3}k_{-2})}{(Tk_2 + k_3 + k_{-2} + k_{-3})} + pf_{fre□}(Tk_1 - k_{-1})$$

The first term on the right-hand side corresponds to the Alp14-facilitated pathway, the second corresponds to the Alp14-independent pathway, with $pf_{free}$ the fraction of protofilaments without bound Alp14. This final term is exactly 1 for a model in which a given protofilament can simultaneously undergo Alp14 mediated and Alp14 independent GTP tubulin exchange; the resulting fit parameters remain almost unchanged ($k_3$ differs by approximately 10%).

How many Alp14 are bound to a microtubule tip? First, note that the data (Figure 4C, main text) require some Alp14 acceleration at Alp14 concentrations as low as 6 nM. This sets an upper limit on the affinity of Alp14 for microtubules of roughly $K_{D,\,Alp14\text{-}MT} \sim 6$ nM in order for at least a single Alp14 to be bound to the microtubule. On the other hand, 0.5 $\mu$M tubulin induces microtubule growth of 12 nm $s^{-1}$ in the presence of Alp14, which requires a $K_{D,\,Alp14\text{-}MT} > 5$ nM given the constraint of fitting to the Alp14-facilitated growth data of Figure 5A, main text. At lower $K_D$ values, the fit parameters for $k_{\pm 2}$ and $k_{\pm 3}$ to the 6 $\mu$M tubulin data cannot induce growth fast enough at 0.5 $\mu$M tubulin. Thus, the affinity of Alp14 for the microtubule tip must be strong, $K_{D,\,Alp14\text{-}MT} \approx 6$ nM.

How do the experimental data constrain the rates $k_{\pm 2}$ and $k_{\pm 3}$? $k_3$ is constrained by the linear effect of Alp14 concentration on microtubule growth at a constant tubulin concentration (Figure 3). This results in $k_3 \approx 6\ s^{-1}$; deviations of more than 20% cannot be compensated by altering $k_{\pm 2}$ and $k_{-3}$.

On the other hand, $k_{\pm 2}$ and $k_{-3}$ are interdependent in fitting the growth rate. As mentioned above, the constraints are growth as a function of Alp14 concentration at 6 $\mu$M tubulin, a maximum growth rate of ~ 35 nm/s at 6 $\mu$M tubulin (presumed to correspond to fully loading the MT tip with Alp14), and a minimum growth rate of 12 nm/s at 0.5 $\mu$M tubulin for a fully loaded MT tip (that is, 13 Alp14 per microtubule plus end). The one constraining parameter is $K_{crit,\,2}$, the critical concentration of tubulin for microtubule growth in the presence of Alp14. This is ~0.08 $\mu$M, a 15-fold reduction from the 1.2 $\mu$M critical concentration for *S. pombe* tubulin without Alp14.

Thus, these three parameters ($k_{\pm 2}$ and $k_{-3}$) are under-constrained. Assuming $k_2 = 50\ \mu M^{-1}\ s^{-1}$ (just about the minimum of 40 $\mu M^{-1}\ s^{-1}$ leads to a range of values for $k_{-2}$ and $k_{-3}$ of ~ 1 to 15 $s^{-1}$, with the two values inversely related.

The final point of comparison is microtubule catastrophe. There are competing models for the details of microtubule catastrophe, but the consensus is that catastrophe follows breaching of the GTP-tubulin cap at the plus end of the microtubule. To be consistent with the increased catastrophe at low tubulin concentrations, then, Alp14 must *increase* the effect of cap breaches at a low solution tubulin concentration (0.5 $\mu$M tubulin shows a high catastrophe rate), but *decrease* the effect of cap breaches at a high solution tubulin concentration (6 $\mu$M tubulin shows a decreasing catastrophe rate with increasing Alp14 concentration).

The model predicts the rate of GTP-tubulin removal required to create a (single protofilament) gap at the end of the microtubule, the lifetime of such gaps, and the average number of gaps per microtubule tip under a given set of conditions. The quantitative predictions depend on the specific $k_{\pm 2}$ and $k_{\pm 3}$ values chosen, but the qualitative results remain consistent across the range of $k_{\pm 2}$ and $k_{\pm 3}$ values discussed above. Sample predictions are shown in Figures S1 below.

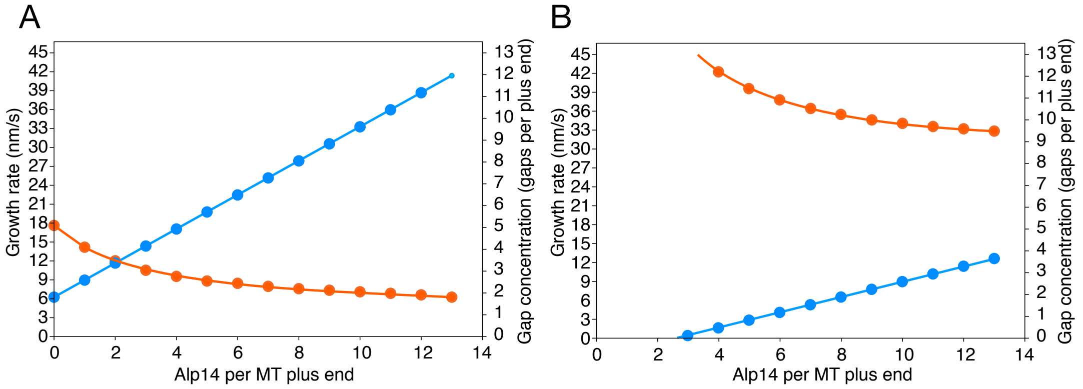


**Figure S1.** (**A**) Growth rate and gap concentration as a function of Alp14s per MT plus end at 6 $\mu$M tubulin. The blue line indicates the growth rate, the orange the number of gaps per microtubule end. At a growth rate of 12 nm $s^{-1}$ (one experimental condition), the model predicts an average of two Alp14 enzymes per plus tip and 3-4 gaps per plus tip. Under these conditions, the lifetime of the microtubules is roughly 250 s. Moreover, the number of gaps decreases with increasing growth rate, consistent with the decreased catastrophe rate of Figure 5A, main text. (**B**) Growth rate and gap concentration as a function of Alp14s per MT plus end at 0.5 $\mu$M tubulin. At 12 nm $s^{-1}$ growth, the model predicts full occupancy of the MT plus end (13 Alp14s) and an average of 9-10 gaps. Under these conditions, the lifetime of the microtubules is roughly 40 s.

As shown in Figures 5 and 6, with $k_{-2} = 3\ s^{-1}$ and $k_{-3} = 9\ s^{-1}$, the number of gaps increases 3-fold going from high to low tubulin concentrations. Alternate combinations of $k_{-2}$ and $k_{-3}$ give a range of gap increases from 2-fold (when the off-rate is limited by a low $k_{-3}$) to 3-fold (when the off-rate is limited by a low $k_{-2}$). Thus, under all parameter sets, the model predicts that the average number of gaps increases in the presence of Alp14. However, the magnitude of this increase is less than the increase in catastrophe rate (~ 6-fold), suggesting that catastrophe depends on cooperative breaching of the GTP cap (either gaps deeper than a single subunit [2], more than one gap [3], or changing tip structure [4].

## Supplementary References

1. Walker RA*, et al*. Dynamic instability of individual microtubules analyzed by video light microscopy: rate constants and transition frequencies. *The Journal of cell biology* **107**, 1437-1448 (1988).

2. Li X, Kolomeisky AB. Theoretical analysis of microtubule dynamics at all times. *J Phys Chem B* **118**, 13777-13784 (2014).

3. Bowne-Anderson H, Zanic M, Kauer M, Howard J. Microtubule dynamic instability: a new model with coupled GTP hydrolysis and multistep catastrophe. *BioEssays : news and reviews in molecular, cellular and developmental biology* **35**, 452-461 (2013).

4. Gardner MK, Charlebois BD, Janosi IM, Howard J, Hunt AJ, Odde DJ. Rapid microtubule self-assembly kinetics. *Cell* **146**, 582-592 (2011).

| Strain | Genotype | Shorthand |
|---|---|---|
| MS1621* | *h*⁻ *alp*7::*ura*4⁺ GFP-*atb2*-*kan*ʳ *leu1*.32 *ura4d18* | *alp*7 deletion |
| MS1618* | *h*⁻ *alp*14::*ura*4⁺ GFP-*atb2*-*kan*ʳ *leu*1.32 *ura4d18* | *alp14* deletion |
| MS1386* | *h*⁻ GFP-*atb2*-*kan*ʳ *leu1*.32 *ura4d18* | Control |
| 972 | *h*⁻ | Dual isoform |
| mmsp174** | *h*⁻ *ura4.d18 arg3.D4 atb2*⁻*::nda2*⁺ | Single isoform |

**Table S2**

***S. pombe* strains used in this study.**

* Strains used in Sato et al. (2004) with GFP-tubulin fusions. Strains were a kind gift from Takashi Toda
** Braun et al (2009)

| Temperature °C | Strain | Growth rate $\mu$m min$^{-1}$ | Shrinkage rate $\mu$m min$^{-1}$ |
|---|---|---|---|
| 26.5 | *alp7* deletion | 1.78 ± 0.11 (12) | 4.17 ± 0.31 (10) |
| | *alp14* deletion | 1.17 ± 0.08 (6) | 4.32 ± 0.44 (7) |
| | control | 3.00 ± 0.19 (8) | 10.16 ± 0.52 (11) |
| 35 | *alp7* deletion | 1.80 ± 0.36 (7) | 4.68 ± 1.08 (6) |
| | *alp14* deletion | 1.98 ± 0.08 (12) | 4.13 ± 0.63 (6) |
| | control | 4.34 ± 0.19 (20) | 13.71 ± 0.87 (19) |

**Table S3**

**Growth and shrinkage rates of *S. pombe* IMAs** at permissive (26.5 °C) and restrictive (35 °C) temperatures. Mean ± SEM (*n*).

| Strain | *n* | Growth % | Shrinkage % | Pause % |
|---|---|---|---|---|
| *alp7* deletion | 7 | 42.8 ± 4.6 | 29.5 ± 2.1 | 27.7 ± 6.2 |
| *alp14* deletion | 8 | 21.4 ± 8.2 | 13.1 ± 3.7 | 65.5 ± 11.5 |
| control | 7 | 49.1 ± 3.5 | 30.8 ± 2.7 | 20.1 ± 4.8 |

**Table S4**

**Percentage of time spent in each phase of MT dynamics.**

Mean ± SEM; *n*, number of cells.

## Legends to Supplementary Movies

**Supplementary Movie 1**
Dynamics of GFP-labeled Interphase Microtubule Arrays (IMAs) in Wild type (left), *alp14* deletion (centre) and *alp7* deletion (right) strains of *S. pombe*.

**Supplementary Movie 2**
Darkfield microscopy of the *in vitro* dynamics of *S. pombe* MTs, built from single isoform (α1β) tubulin, with and without added 12 nM Alp14. Images of fluorescently labeled stabilised MT seeds (green) are overlaid.

**Supplementary Movie 3**
Darkfield microscopy of the effect of added Alp7 on Alp14-catalysed *in vitro* dynamics of *S. pombe* MTs. Extraordinarily longlived, fast-growing microtubules appear (right). Images of fluorescently labeled stabilised MT seeds (green) are overlaid.

**Supplementary Movie 4**
Dual colour TIRF microscopy. Effect of Alp7 on tip tracking by Alp14. In the sequence at left, tip tracking by Alp14 is partially suppressed by added brain tubulin (see main text). Addition of Alp7 restores tip tracking (white arrowheads). Growth rate increases only slightly, consistent with continued inhibition by brain tubulin.
